# Loss of NAMPT in aging retinal pigment epithelium reduces NAD^+^ availability and promotes cellular senescence

**DOI:** 10.18632/aging.101469

**Published:** 2018-06-12

**Authors:** Ravirajsinh N. Jadeja, Folami L. Powell, Malita A. Jones, Jasmine Fuller, Ethan Joseph, Menaka C. Thounaojam, Manuela Bartoli, Pamela M. Martin

**Affiliations:** 1 Department of Biochemistry and Molecular Biology, Medical College of Georgia at Augusta University, Augusta, GA 30912, USA; 2 Education Innovation Institute, Medical College of Georgia at Augusta University, Augusta, GA 30912, USA; 3 James and Jean Culver Vision Discovery Institute, Medical College of Georgia at Augusta University, Augusta, GA 30912, USA; 4 Department of Ophthalmology, Medical College of Georgia at Augusta University, Augusta, GA 30912, USA; 5 Georgia Cancer Center, Medical College of Georgia at Augusta University, Augusta, GA 30912, USA

**Keywords:** retinal pigment epithelium (RPE), aging, age-related macular degeneration (AMD), NAD+, NAMPT, senescence, SIRT1

## Abstract

Retinal pigment epithelium (RPE) performs numerous functions critical to retinal health and visual function. RPE senescence is a hallmark of aging and degenerative retinal disease development. Here, we evaluated the temporal expression of key nicotinamide adenine dinucleotide (NAD^+^)-biosynthetic genes and associated levels of NAD^+^, a principal regulator of energy metabolism and cellular fate, in mouse RPE. NAD^+^ levels declined with age and correlated directly with decreased nicotinamide phosphoribosyltransferase (NAMPT) expression, increased expression of senescence markers (p16^INK4a^, p21^Waf/Cip1^, ApoJ, CTGF and β-galactosidase) and significant reductions in SIRT1 expression and activity. We simulated *in vitro* the age-dependent decline in NAD^+^ and the related increase in RPE senescence in human (ARPE-19) and mouse primary RPE using the NAMPT inhibitor FK866 and demonstrated the positive impact of NAD^+^-enhancing therapies on RPE cell viability. This, we confirmed *in vivo* in the RPE of mice injected sub-retinally with FK866 in the presence or absence of nicotinamide mononucleotide*.* Our data confirm the importance of NAD^+^ to RPE cell biology normally and in aging and demonstrate the potential utility of therapies targeting NAMPT and NAD^+^ biosynthesis to prevent or alleviate consequences of RPE senescence in aging and/or degenerative retinal diseases in which RPE dysfunction is a crucial element.

## Introduction

The retinal pigment epithelium (RPE) performs numerous functions essential to normal retinal health and function [1]. RPE serves as a physiologic barrier between the photoreceptor cells and the choroidal blood supply and in doing so, plays an essential role in protecting the retina from systemic insults by regulating immune responses and thereby limiting the entry of infectious or otherwise detrimental agents into retina [1]. Additionally and importantly, RPE facilitates the delivery of nutrients and ions extracted from the choroidal blood to the photoreceptors and the counter movement of photoreceptor-derived waste products for disposition in the choroid [2]. As such, damage to or dysfunction of RPE may have a domino effect on the health and function of photoreceptors that are obligatorily dependent upon it and therefore a significant impact on visual function. Hence, it is not surprising that RPE is implicated directly and prominently in the pathogenesis of most degenerative diseases of the retina, including age-related macular degeneration (AMD), the leading cause of blindness among persons aged 60 and above worldwide [3,4].

AMD is a complex multifactorial disease and, as its name implies, age is a primary risk factor for its development [5]. RPE is principally affected in AMD [6]. Interestingly however, most available experimental models and related studies, including our own prior, have focused more heavily on identifying, understanding and limiting secondary consequences of aging and related RPE dysfunction (e.g., increased oxidative stress, inflammation, altered cholesterol metabolism) as opposed to targeting directly factors, such as energy deprivation, that precipitate accelerated aging at a cellular level. The consequence of the latter is an imbalance in homeostatic processes and subsequent damage, as shown in many specific cell types. This is the premise of a number of recent studies including the present investigation in which we focused on nicotinamide adenine dinucleotide (NAD^+^) and factors governing its bioavailability in relation to the overall impact on RPE viability.

NAD^+^, a central metabolic cofactor, plays a critical role in regulating cellular metabolism and energy homeostasis [7,8]. The ratio of NAD^+^ to NADH (oxidized to reduced NAD^+^) regulates the activity of various enzymes essential to metabolic pathways including glycolysis, the Kreb’s cycle, and fatty acid oxidation [8]. There is a wealth of clinical and experimental data stemming from studies of other primary diseases of aging (e.g., Alzheimer’s disease, type 2 diabetes, non-alcoholic fatty liver disease, etc.) demonstrating clearly a generalized decline in the availability of NAD^+^ in association with increased age and the related reduction in the activity of a number of downstream metabolic pathways that contribute to the development and progression of degenerative processes [7,8]. Neuronal cells and tissues appear to be especially sensitive in this regard. Importantly, the aforementioned studies additionally suggest that age-related degenerative processes might be prevented or at the least, the consequences thereof lessened in severity by therapies that boost NAD^+^. The expression and activity of a number of key NAD^+^-dependent proteins (e.g., members of the sirtuin family, poly ADP-ribose polymerases (PARPs) and cyclic ADP-ribose synthases) and, the efficacy of therapies capable of impacting them have been evaluated in the context of aging retina and RPE [9–11]. However, little attention has been given to upstream factors that regulate NAD^+^ biosynthesis, particularly in RPE. Given the importance of RPE to retinal health and function, in the present investigation we focused on evaluating the impact of NAD^+^ and factors that regulate its availability on RPE viability both *in vivo* and *in vitro.* Collectively, our results confirm the importance of NAD^+^ to RPE health and the positive potential impact of therapies that enhance NAD^+^ generation in this cell type hold with respect to limiting senescence and preserving RPE viability. This finding is highly relevant to the clinical management of AMD but perhaps also broadly to the management of other degenerative retinal diseases in which RPE is prominently affected.

## RESULTS

### NAD^+^ levels decline with increasing age in mouse RPE

**Figure 1:**
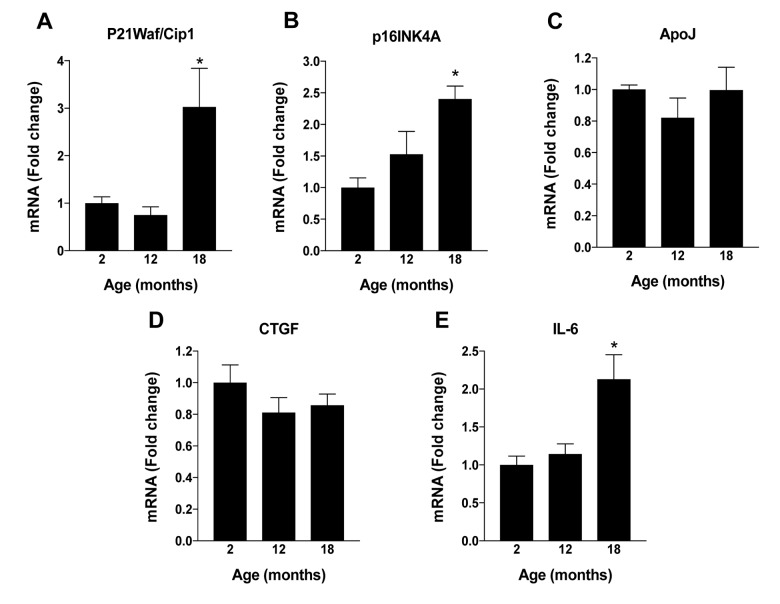
**Changes in RPE NAD+ metabolism with aging in mice.** RPE/eye cup was dissected from 2, 12 and 18 months old male C57BL/6J mice to evaluate changes in NAD^+^ metabolism. (**A**) NAD^+^ content was measured using a commercially available kit. (**B**) Overview of NAD synthesis pathways in mammals. (**C**-**E**) Changes in mRNA expression of enzymes regulating NAD^+^ synthesis were performed by qPCR. (**F**) Changes in SIRT-1 protein levels were measured by western blotting. Data is presented as mean ± S.E.M for n=5. A representative western blot image from three replicates is shown. mRNA expression of genes were normalized to 18s expression. *p<0.05 compared to 2M (two months old mice). NAMPT; Nicotinamide phosphoribosyltransferase, QPRT; Quinolinate Phosphoribosyltransferase, NMNAT; Nicotinamide mononucleotide adenylyltransferease.

NAD^+^ levels decline with age in many cell and tissue types [12,13]. Whether the same occurs in aging RPE is unclear. As such, our first step was to evaluate temporally the availability of NAD^+^ in the RPE of the living animal. To do so, we isolated RPE tissue from the eyes of C57BL/6J mice that per the established average lifespan of a normal lab mouse (~24 months) belonged to one of the following three age groups: young-adult (age 1.5 - 6 months), middle-aged adult (age 7 – 11 months) or adults of advanced age (12 months of age or greater). Levels of NAD^+^ in mouse RPE correlated inversely with age (Fig. 1A). NAD^+^ levels remained stable in this cellular layer up to six months of age but began to decline significantly shortly thereafter such that by 12 months of age, there was on average, a 40-50 percent decrease in RPE-specific NAD^+^ content. Further reductions in NAD^+^ were detected in RPE obtained from mice of advanced age.

### Nicotinamide phosphoribosyltransferase (NAMPT) is essential to NAD^+^ and down-stream SIRT1 expression in RPE

To identify the specific mechanism(s) responsible for the age-dependent decline in levels of NAD^+^ that we observed in mouse RPE, we next evaluated the expression of enzymes pertinent to NAD^+^ biosynthesis. In mammals, NAD^+^ is generated via one of two principal routes, the *de novo* or the salvage pathway ([8,14]; Fig. 1B). Congruent with this, we monitored the expression of nicotinamide adenylyltransferase (NMNAT), quinolinate phosphoribosyltransferase (QPRT) and nicotinamide phosphoribosyltransferase (NAMPT), key enzymes relevant to one or both of the aforementioned major pathways to NAD^+^ generation. NMNAT and QPRT expression in RPE remained relatively stable across all ages tested despite the age-associated decline in NAD^+^ content (Figs. 1C and D, respectively). However, NAMPT expression (Fig. 1E) mirrored closely the age-dependent decline in NAD^+^, strongly implicating this enzyme as the principal enzyme driving NAD^+^ generation in RPE. This finding is consistent with findings in the existing scientific literature demonstrating that the bulk of NAD^+^ that is generated in mammals under physiological conditions is derived from the salvage pathway, also known as the deamidated route [14]. We evaluated also the expression of sirtuin family member 1 (SIRT1), a highly studied NAD^+^-dependent histone deacetylase touted as being a principal metabolic sensor and regulator of cellular energy metabolism and stress responses. Importantly, SIRT1 has also been linked to ocular aging and/or processes associated with the development and progression of age-related retinal disease [15]. Like NAD^+^ and NAMPT mRNA expression, levels of SIRT1 protein declined significantly in mouse RPE in association with increasing age (Fig. 1F). Further, some of the senescence-associated markers were increased in aged mouse RPE (Fig. S1).

### FK866 inhibits NAMPT thereby decreasing NAD^+^ levels and promoting RPE senescence

Our data to this point confirm that NAD^+^ levels decline in RPE as age increases. They also demonstrate the critical importance of NAMPT to the generation and maintenance of NAD^+^ levels in RPE. Hence, therapies targeting NAMPT and NAD^+^ may be of benefit in preserving RPE health and viability in aging and age-related retinal diseases like AMD. However, the development and testing of new therapies in this field of research is impeded significantly by the lack of experimental model systems that recapitulate accurately characteristics of human aging and age-related disease and/or, the extended time periods often required to generate reliable models and conduct such studies. Thus, to further our present studies and evaluate the impact of reduced NAMPT and consequent NAD^+^ availability on RPE cell viability, we used the highly specific and non-competitive NAMPT-inhibitor FK866 [16] to mimic acutely the age-related effect of decreased NAMPT expression and NAD^+^ content in cultured RPE cells and intact mouse retina.

**Figure 2:**
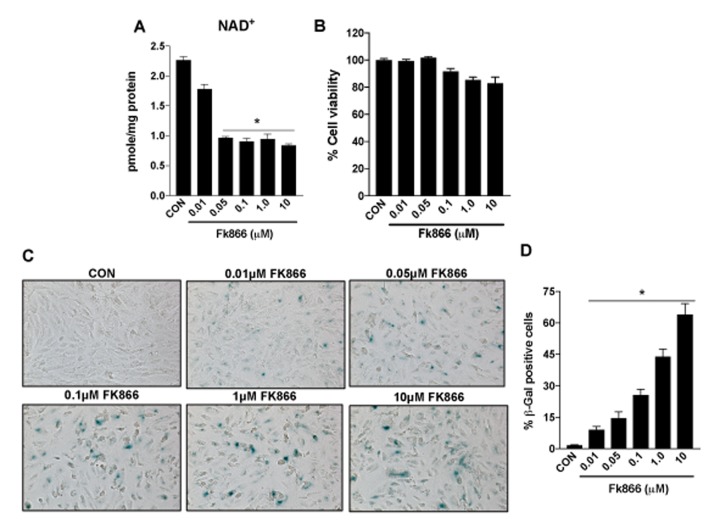
**Inhibition of NAMPT activity in human retinal pigment epithelial cells decreases NAD+ levels to induce senescence.** Human retinal pigment epithelial cells (ARPE-19) were treated with different doses (0.01-10μM) of a selective NAMPT activity blocker, FK866. Dose-dependent changes in (**A**) NAD^+^ content (**B**) cell viability and (**C**-**D**) senescence of FK866 treated human RPE cells were evaluated. Data are presented as mean ± S.E.M for n=3 independent experiments. *p<0.05 compared to CON (Vehicle treated).

**Figure 3:**
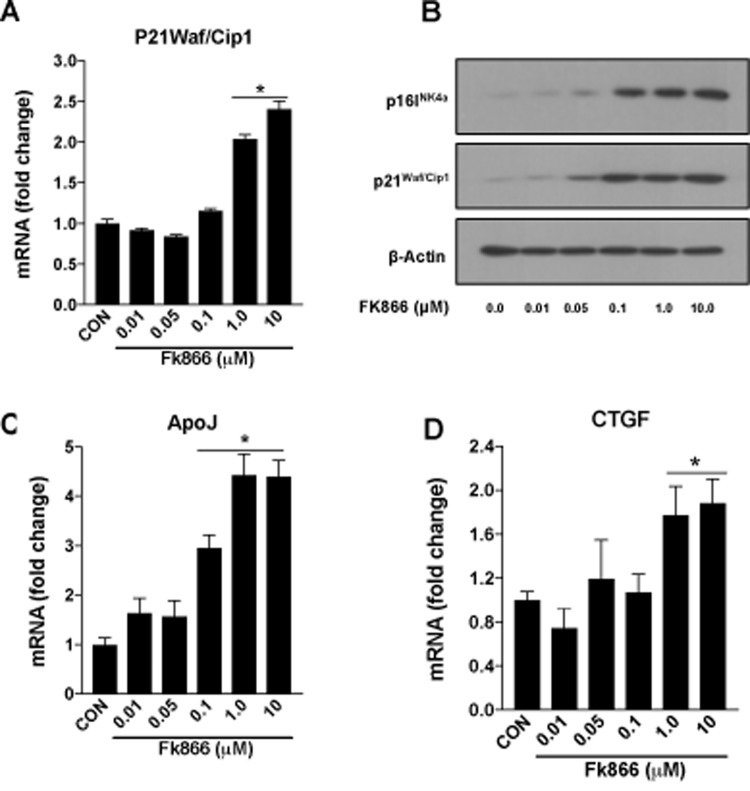
**FK866 treatments induce changes in the markers of senescence in human retinal pigment epithelial cells.** Human retinal pigment epithelial cells (ARPE-19) were treated with different doses (0.01-10μM) of FK866 and changes in the expression of various senescence markers were evaluated by qPCR and western blotting. Dose-dependent changes in (**A**) p21^Waf/Cip1^ mRNA, (**B**) p16^INK4a^ and p21^Waf/Cip1^ protein and, (**C**-**D**) CTGF and ApoJ mRNA levels are shown. A representative western blot image from three replicates is shown. mRNA expression of genes were normalized to 18s expression. Data are presented as mean ± S.E.M for n=3 independent experiments. *p<0.05 compared to CON (Vehicle treated).

**Figure 4:**
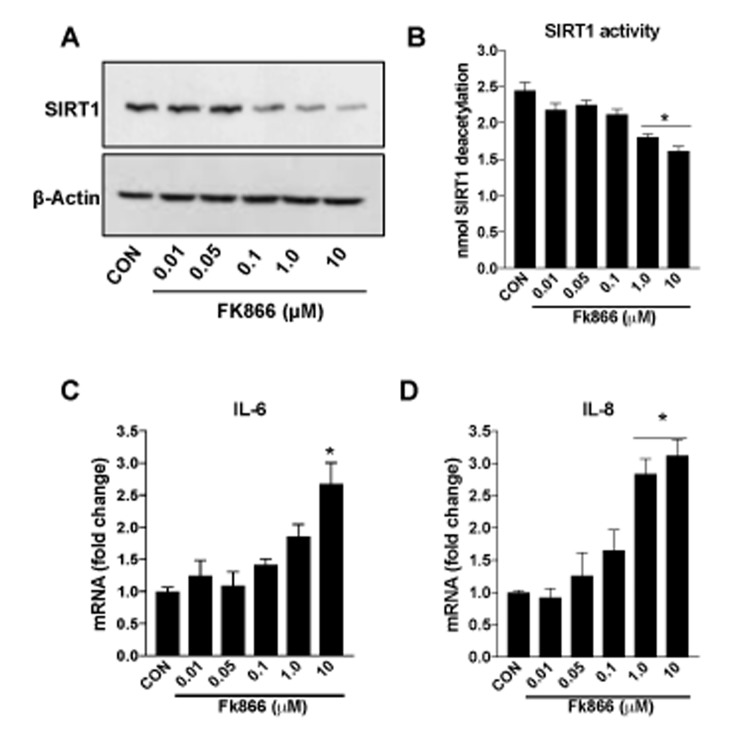
**FK866 decreases SIRT-1 expression/activity and increases inflammation in human retinal pigment epithelial cells.** Human retinal pigment epithelial cells (ARPE-19) were treated with different doses (0.01-10μM) of FK866 and (**A**) expression and (**B**) activity of SIRT1 was evaluated by western blotting and commercially available SIRT1 assay kit respectively. (**C**-**D**) Changes in inflammatory markers (IL-6 and IL-8) were evaluated by qPCR. A representative western blot image from three replicates is shown. mRNA expression of genes were normalized to 18s expression. Data are presented as mean ± S.E.M for n=3 independent experiments. *p<0.05 compared to CON (Vehicle treated).

Confluent, well-differentiated cultures of human RPE (ARPE-19) cells were exposed to varying concentrations of FK866 for 72 h. As anticipated, FK866 induced a dose-dependent decline in levels of NAD^+^ (Fig. 2A). Cell viability assays confirmed the absence of robust cellular toxicity due to FK866 exposure across the range of doses of the compound that were tested (0.01 – 10 μM; Fig. 2B). There was a significant and dose-dependent increase in the number of RPE cells positive for the expression of β-galactosidase, an established marker of cellular senescence, in cells treated with FK866 (Fig. 2C-D), suggesting that while the compound did not induce robust cell death, it did on the other hand, stimulate cellular senescence. To confirm this, we evaluated also the mRNA and/or protein expression of cyclin-dependent kinase inhibitor 1 (p21^Waf/Cip1^), cyclin-dependent kinase Inhibitor 2A (p16^INK4a^), apolipoprotein J (ApoJ), and connective tissue growth factor (CTGF), key additional biomarkers of aging and cellular senescence [17–20]. Congruent with β-galactosidase staining, the expression of each of these markers was increased in association with increasing concentrations of FK866 and the associated decline in NAD^+^ (Fig. 3A-D). Additionally, the expression and activity of SIRT1 was dose-dependently decreased (Fig. 4A-B).

A common consequence of cellular senescence is the decreased ability of cells to handle the oxidative stress produced as a normal consequence of cellular metabolism [21]. This potentiates inflammatory processes and the further production of pro-oxidant byproducts. This vicious cycle of oxidative stress and inflammation has been identified as key factor in the development and progression of AMD and other degenerative retinal diseases. As such, we evaluated the effect of FK866 on the expression of interleukins 6 and 8 (IL-6 and IL-8, respectively), two pro-inflammatory cytokines that have been shown to be elevated commonly in degenerating RPE [17,22]. Levels of IL-6 and IL-8 were increased in association with increasing concentrations of FK866 in our experimental system (Fig. 4C-D).

**Figure 5:**
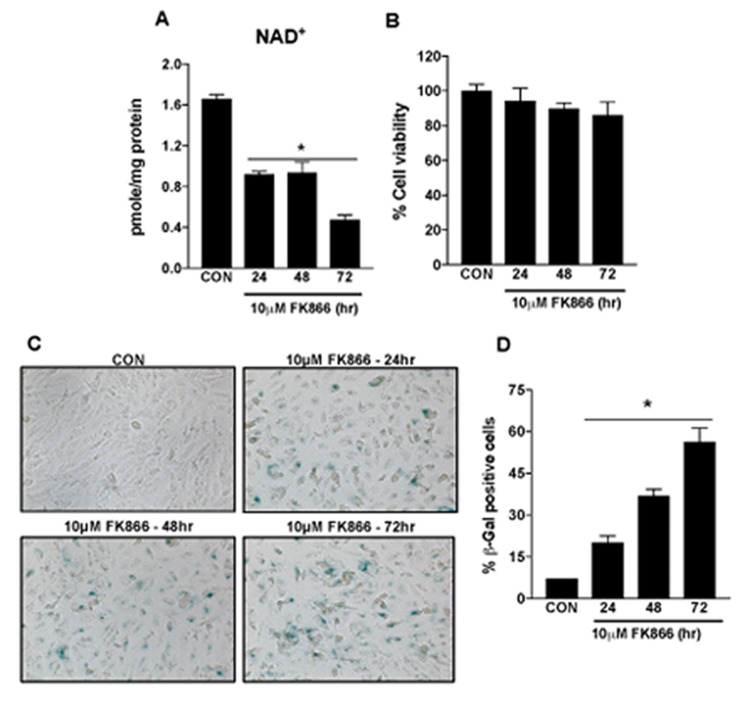
**Time-dependent decline in NAD+ content and induction of senescence in human retinal pigment epithelial cells treated with FK866.** Human retinal pigment epithelial cells (ARPE-19) were treated with 10μM FK866 for 24, 48 and 72 hr. Time-dependent changes in (**A**) NAD^+^ content (**B**) cell viability and (**C**-**D**) senescence of FK866 treated human RPE cells were evaluated. Data are presented as mean ± S.E.M for n=3 independent experiments. *p<0.05 compared to CON (Vehicle treated).

**Figure 6:**
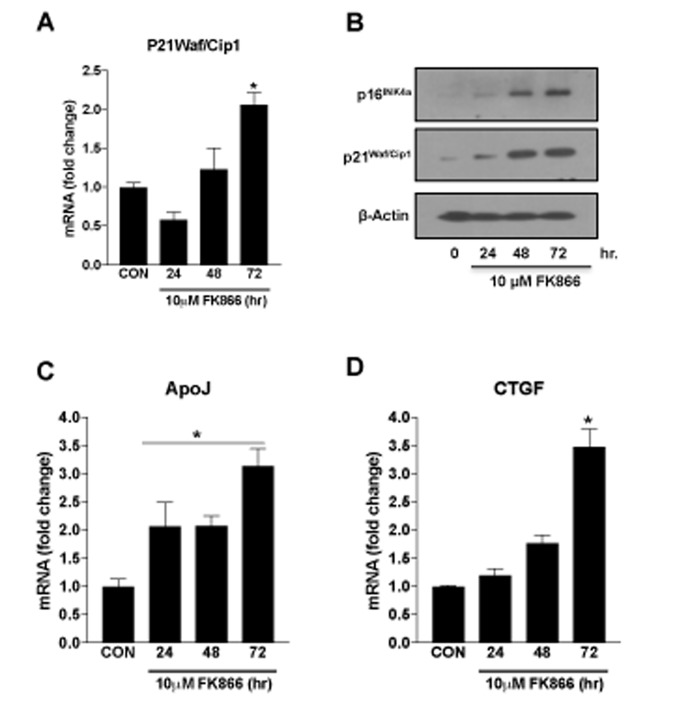
**Time-dependent changes in the markers of senescence in human retinal pigment epithelial cells treated with FK866.** Human retinal pigment epithelial cells (ARPE-19) were treated with 10μM FK866 for 24, 48 and 72 hr. and changes in the expression of various senescence markers was evaluated by qPCR and western blotting. Time-dependent changes in (**A**) p21^Waf/Cip1^ mRNA, (**B**) p16^INK4a^ and p21^Waf/Cip1^ protein and, (**C**-**D**) CTGF and ApoJ mRNA levels are shown. A representative western blot image from three replicates is shown. mRNA expression of genes were normalized to 18s expression. Data are presented as mean ± S.E.M for n=3 independent experiments. *p<0.05 compared to CON (Vehicle treated).

**Figure 7:**
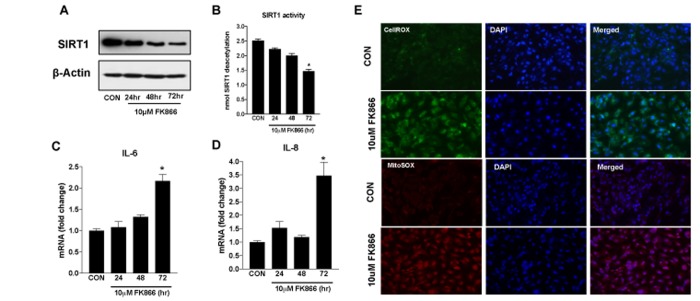
**Time-dependent changes in SIRT-1 expression/activity and inflammatory markers in human retinal pigment epithelial cells treated with FK866.** Human retinal pigment epithelial cells (ARPE-19) were treated with 10μM FK866 for 24, 48 and 72 hr. and (**A**) expression and (**B**) activity of SIRT-1 was evaluated by western blotting, and commercially available SIRT-1 assay kit respectively. (**C**-**D**) Changes in inflammatory markers (IL-6 and IL-8) were evaluated by qPCR. (**E**) Representative images of CellROX and MitoSOX stained 10μM FK866 treated (72 hr.) A representative western blot image from three replicates is shown. mRNA expression of genes were normalized to 18s expression. Data are presented as mean ± S.E.M for n=3 independent experiments. *p<0.05 compared to CON (Vehicle treated).

Based upon our immediate prior findings, a dose of 10 µM FK866 was confirmed as the optimal standard dose. To confirm the optimal incubation period for FK866 treatment, additional cultures of ARPE-19 were treated with 10 µM FK866 for 24, 48 or 72 h and parameters identical to those described above (Figs. 2-4) were evaluated. FK866 was effective at reducing NAD^+^ levels at each of the time points tested, however the greatest efficacy was achieved in association with a 72 h incubation period (Fig. 5A), the time period that was used in our initial studies. Cell viability assays were repeated in these additional batches of cells and the absence of cellular toxicity in cells exposed to 10 µM FK866 at all-time points tested was confirmed (Fig. 5B). β-galactosidase staining and related quantification revealed a time-dependent increase in the number of β-gal-positive cells in FK866-treated cultures (Fig. 5C-D). Similarly, the expression of p21^Waf/Cip1^, p16^INK4a^, ApoJ and CTGF was increased (Fig. 6A-D) and SIRT1 expression and activity decreased (Fig. 7A-B) time-dependently, further supporting the optimality of the 72 h incubation period (Fig. 6A-D).

### FK866 induces expression of inflammatory factors and ROS production in RPE

Senescent cells secret numerous kinds of pro-inflammatory cytokines, chemokines, growth factors and proteases, exemplifying what is referred to as a senescence-associated secretory phenotype (SASP) [23]. Further, oxidative stress also plays an important role in RPE senescence [24–26]. Congruent with this, we treated additional ARPE-19 cells with 10 µM FK866 for 72 h, the dose and time-point most effective at reducing NAD^+^ and promoting cellular senescence without robust toxicity, and monitored them for expression of the pro-inflammatory cytokines IL-6 and IL-8 (Fig. 7C-D), and CellROX and MitoSOX positivity, a general marker of oxidative stress and a mitochondrial-specific superoxide marker, respectively (Fig. 7E). Like IL-6 and IL-8 expression, the number of cells positively stained with CellROX or MitoSOX dyes (green fluorescence and red fluorescence, respectively) and therefore in which there was an increased amount of oxidative stress, was increased in ARPE-19 cells exposed to FK866 compared to control, non-exposed cell cultures (Fig. 7E).

### Nicotinamide mononucleotide (NMN) treatment preserves NAD^+^ and prevents RPE senescence *in vitro*

**Figure 8:**
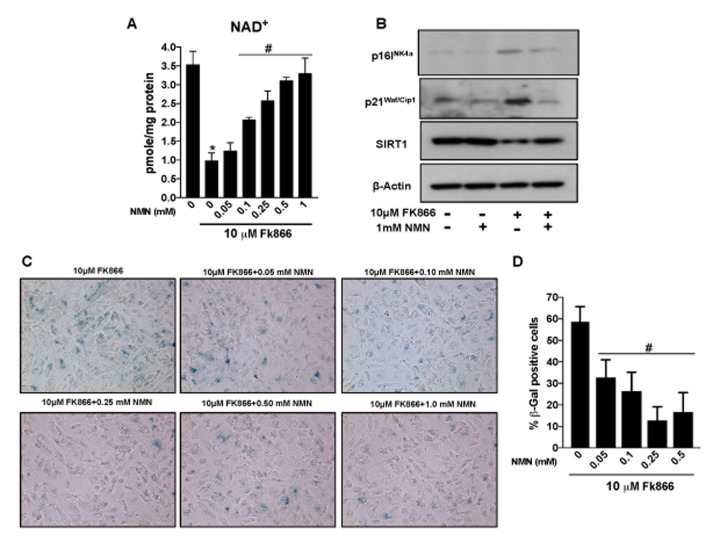
**Nicotinamide mononucleotide (NMN)treatment preserves NAD^+^ and prevents senescence in human retinal pigment epithelial cells.** Human retinal pigment epithelial cells (ARPE-19) were treated with 10μM FK866 alone or in combination with different doses of NMN (0.05-1 mM) for 72 hr. (**A**) NAD^+^ content was measured using a NAD assay kit. (**B**) Changes in expression of SIRT1, p16^INK4a^ and p21^Waf/Cip1^ proteins levels were evaluated by western blotting. (**C**-**D**) RPE senescence was evaluated by β-galactosidase staining. A representative western blot image from three replicates is shown. Data are presented as mean ± S.E.M for n=3 independent experiments. *p<0.05 compared to CON (Vehicle treated) and #p<0.05 compared to FK866.

Therapies that enhance NAD^+^ have been shown to reduce the incidence and/or severity of age-associated complications [27–31]. However, it is unclear, whether the latter is also of benefit in aged RPE and associated degenerative retinal disease. Our studies up to this point have established the decline in NAMPT and related NAD^+^ availability in aged RPE and validated the FK866-treated cell culture system as a suitable *in vitro* model system in which to study these factors. Therefore, we used this experimental model to determine whether augmenting NAD^+^ content using the NAD^+^ precursor nicotinamide mononucleotide (NMN) could preserve NAD^+^ and prevent FK866-induced RPE senescence. NMN dose-dependently enhanced NAD^+^ levels in cells exposed to FK866 (Fig. 8A). Additionally, the compound prevented FK866-induced RPE senescence as indicated by the suppression of p16^INK4a^ and p21^Waf/Cip1^ expression and the reduction of SIRT1 expression (Fig. 8B). The number of β-galactosidase positive cells was also decreased in cultures exposed to NMN (Fig. 8C-D).

**Figure 9:**
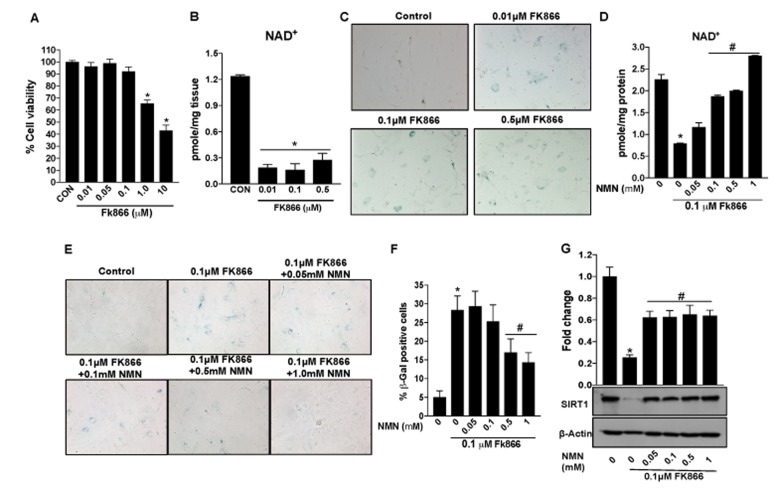
**Nicotinamide mononucleotide (NMN)treatment preserves NAD^+^ and prevents senescence in mouse retinal pigment epithelial cells.** Primary RPE cells were isolated from 17 days old mouse pups and cultured as described in materials and methods. Mouse primary RPE cells were treated with different doses of FK866 for 5 days to evaluate changes in cell viability, NAD^+^ content, senescence and SIRT1 expression. (**A**) Cell viability was evaluated by MTT assay. (**B** and **D**) NAD^+^ content was measured using a NAD assay kit. (**C**, **E** and **F**) RPE senescence were evaluated by and β-galactosidase staining. (**G**) Changes in expression of SIRT-1 protein levels were evaluated by western blotting. A representative western blot image from three replicates is shown. Data are presented as mean ± S.E.M for n=3 independent experiments. *p<0.05 compared to CON (Vehicle treated) and #p<0.05 compared to FK866.

ARPE-19, though of human origin, is a “transformed” retinal cell line. Therefore, to validate findings obtained using ARPE-19 cell cultures, key experiments were repeated using cultures of primary mouse RPE (Fig. 9). FK866 was effective at reducing NAD^+^ expression in primary RPE though cell viability data indicate increased sensitivity in the form of cellular toxicity (Fig. 9A). Therefore, in subsequent studies using these cells, we refrained from using higher doses of the compound (Fig. 9B-G). As observed in ARPE-19 cells, exposure of RPE cells to FK866 potentiated cellular senescence (Fig. 9C), and these phenomena were inhibited by co-exposure of cells to NMN (Fig. 9D-F). Importantly, NMN also prevented the FK866-induced decrease in SIRT1 expression (Fig. 9G).

### Nicotinamide mononucleotide (NMN)treatment preserves NAD^+^ and prevents RPE senescence *in vivo*

**Figure 10:**
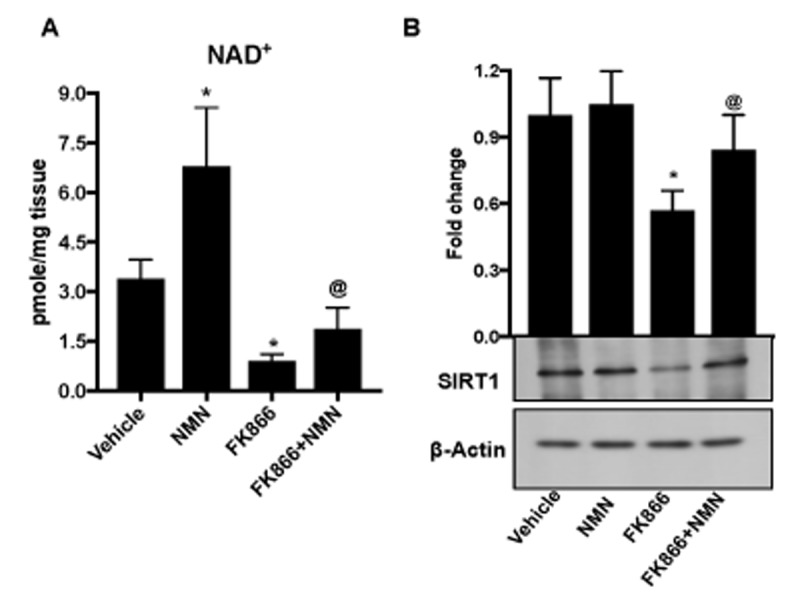
***In vivo* administration of nicotinamide mononucleotide (NMN) prevents FK866-induced NAD^+^ depletion and RPE senescence.** Male C57BL/6J mice were injected sub-retinally with 10 μM FK866 (right eye) at day 0 and day 7 and sacrificed on day 15 (7 days after the last dose). RPE/eye cup was then collected for further analysis. Simultaneously, a group of mice were treated with 150 mg/kg NMN (*i.p.*) for 14 days. Left eye was injected with PBS to use as controls. (**A**) NAD^+^ content and (**B**) SIRT-1 expression was evaluated by NAD assay and western blotting respectively. A representative western blot image from three replicates is shown. Data are presented as mean ± S.E.M for n=5. *p<0.05 compared to CON (Vehicle treated) and @p<0.05 compared to FK866.

To determine whether our findings in the ARPE-19 and primary RPE cell culture model systems could be extrapolated to the living animal, we injected FK866 sub-retinally into the eyes and/or NMN intraperitoneally to adult C57BL/6J mice. NAD^+^ levels were significantly lower in RPE tissue isolated from mice injected with FK866 than in vehicle (PBS)-injected controls (Fig. 10A). On the other hand, NAD^+^ levels more than doubled in association with NMN injection only, and in animals that received NMN in conjunction with FK866, NAD^+^ levels were significantly preserved. SIRT1 expression was additionally preserved in conjunction with NMN injection (Fig. 10B).

## DISCUSSION

RPE performs a number of highly diverse functions that are essential to maintenance of the normal health and function of the retina. Hence, it is not surprising that dysfunction of this cellular layer is implicated causally in the development and progression of a number of degenerative diseases of the retina. Cellular senescence is a common consequence of aging hence, the decline in NAD^+^ in RPE and the associated upregulated expression of markers of senescence that we observed was not totally surprising. Though there has been some debate over whether dysfunction occurs first in the RPE or in the overlying photoreceptors, the contribution of senescence-associated RPE damage to age-related RPE dysfunction is undeniable. Irrespective of views on the exact chronology of the events that initiate degenerative processes in the aging outer retina, the importance of preserving a healthy RPE is emphasized by the fact that there is, to our knowledge, no experimental or clinical condition in which photoreceptors persist unaffected above an underlying region of RPE that is damaged, largely dysfunctional or dead. This is exemplified by the severe irreversible loss of central vision that can occur in association with geographic atrophy or RPE cell dropout in advanced dry AMD. Thus, understanding the mechanisms that govern age-related changes in RPE is both important and essential to the development of therapies to effectively prevent and treat age-related degenerative retinal diseases like AMD. This is especially true regarding the dry form of the disease, which (a) generally presents first, (b) impacts the greatest number of patients, and (c) is not highly amenable to any form of therapeutic management that is presently available [32–34]. With these facts in mind, in the present study we focused on NAD^+^.

NAD^+^ levels decline with age in many cell and tissue types. In fact, altered NAD^+^ metabolism and concurrent alterations in mitochondrial function are inherent in metabolic disorders including type 2 diabetes, non-alcoholic fatty liver disease, and age-related diseases such as Parkinson’s and Alzheimer’s disease [7]. Furthermore, therapies that enhance NAD^+^ production and/or the expression and activity of downstream NAD^+^-dependent enzymes have shown benefit in treating such diseases [35]. In retina specifically, the importance of NAD^+^ normally and in aging, has been evaluated directly in photoreceptors [36] but has not been studied extensively in the underlying RPE. This is both interesting and unfortunate given, as mentioned previously, the numerous essential primary and supportive functions that RPE performs and therefore, the obligatory commensal relationship that exists between the photoreceptors and RPE, the two cell types principally affected in AMD. A very recent report [37], demonstrated, using human-induced pluripotent stem cell derived RPE cells (hiPSC-RPE) prepared from donors with and without AMD, the benefit of the NAD^+^ precursor, nicotinamide, in limiting the expression of key complement and inflammatory proteins linked directly to drusen development and AMD. This study supports the feasibility of targeting NAD^+^ biosynthesis therapeutically to preserve RPE viability and thereby prevent and/or treat related degenerative processes in aged retina.

Here, using adult C57BL/6J mice across a broad range of ages (2 -18 months) and in which the absence of any underlying *rd* mutation that might impact adversely retinal phenotype or function has been verified [38], we first confirmed that NAD^+^ levels decline significantly in association with increased age as has been reported to occur in other retinal and non-retinal cell types. As a positive control, we monitored also the expression of SIRT1, a down-stream NAD^+^-dependent enzyme that is known to decrease in expression in association with increased age and decreased availability of NAD^+^ in various organisms, cell and tissue types. Our related evaluation of enzymes that drive key steps in NAD^+^ biosynthesis revealed NAMPT as the enzyme principally responsible for maintaining adequate NAD^+^ levels in RPE. This is congruent with recent work by others demonstrating that NAMPT-mediated NAD^+^ biosynthesis is essential for proper visual function [36].

A major limiting factor in AMD research is the lack of availability of acute experimental models that mimic accurately processes involved in disease pathogenesis while at the same time, affording a reliable preclinical system in which to rapidly test developmental therapies. FK866 has been used broadly in the cancer field and others to study the impact of NAMPT inhibition on various processes including cellular viability and immune signaling, inflammation, and energy metabolism [16,39,40]. Here, we used the compound to optimize a cell culture model system that allowed us to simulate and study the impact of decreased NAMPT expression and related NAD^+^ availability on RPE cell viability relevant to aging. Our studies in the human RPE cell line ARPE-19 demonstrated an increase in RPE cell senescence in conjunction with reduced NAMPT and NAD^+^ availability as indicated by analyses of the expression of senescence markers like β-galactosidase, ApoJ, p16^INK4a^, p21^Waf/Cip1^ and CTGF. Importantly, co-exposure of cells to the NAD^+^-precursor NMN stabilized levels of NAD^+^ and SIRT1 even in the presence of NAMPT inhibition and prevented the induction of senescence-associated gene expression. ARPE-19 is a transformed cell line and we simulated the age-related decline in NAD^+^ in these cells artificially using FK866. Therefore, we acknowledge that there may be differences between the way these cells respond compared to natural RPE. To alleviate this concern, we repeated key experiments in primary mouse RPE cells. The optimal age for isolating and culturing primary mouse RPE is 18-21 days. Cells can be isolated from mice of older age but do not persist well in culture for extended periods. This makes direct comparative studies between primary RPE cells obtained from young and old mice difficult to standardize. Hence, we again used FK866 to suppress NAMPT activity in primary RPE cells isolated from young mice. Importantly, the validity of our cell culture model systems and the data emanating there from was supported strongly by the fact that findings obtained in our cell cultures mirrored closely those obtained using intact RPE tissue obtained and analyzed immediately upon procurement from the eyes of living animals.

The risk and incidence of disease formation both within and outside of the eye increases proportionally with age. Though the cell and tissue types affected may differ from one disease to another, commonality is found in that the process of aging and the development of age-related degenerative disease generally appears to involve a progressive decline in cellular energy production and consequently, in the viability and function of cells. Neuronal tissues such as retina are especially sensitive. In AMD, gradual degenerative changes occur in both the photoreceptors and the RPE. RPE senescence is thought to be largely responsible for precipitating these changes [41–43]. Some promise with respect to preventing senescence-associated changes in RPE and potentially impacting AMD development has been demonstrated experimentally using compounds like resveratrol, which impact the expression and activity of SIRT1, a down-stream NAD^+^-dependent gene that in turn, governs processes essential to the maintenance of cellular viability [44–46]. However, few have explored directly the potential therapeutic impact of targeting NAMPT and NAD^+^ biosynthesis itself despite the knowledge that NAD^+^ is the starting point of most major metabolic pathways and therefore, the key governor of cellular aging and age-related processes.

Our present data demonstrating an age-dependent decline in NAMPT expression and in turn, NAD^+^ generation in RPE which ultimately promotes RPE senescence supports strongly the rationale for enhancing NAMPT expression and associated NAD^+^ generation therapeutically. Indeed, such therapies may represent a viable strategy for preventing and treating RPE and consequent photoreceptor damage in aging/AMD and broadly, in other degenerative retinal diseases in which RPE is prominently affected. AMD is the leading cause of blindness among persons aged 60 and above worldwide [47,48]. Congruent with advancements in healthcare and the large number of “baby boomers”, the number of persons within this age bracket has and continues to increase steadily and substantially, and proportionate to that, the incidence of AMD. Indeed, it is estimated that by the year 2020, 200 million people will be affected by AMD [48]. Hence, AMD represents a significant present and future global health and economic burden [49], the impact of which is exacerbated by the fact that strategies to prevent and treat dry AMD, the form of the disease that is most common and therefore affects the greatest number of patients, are lacking. Based upon our present experimental observations, future preclinical studies evaluating NMN or other therapies that have a direct impact on NAMPT expression and NAD^+^ metabolism in the context of aging and age-related retinal disease development and progression are highly warranted.

## MATERIALS AND METHODS

### Animals

All experiments involving animals adhered to the Public Health Service Policy on the Humane Care and Use of Laboratory Animals (2015 Department of Health, Education and Welfare publication, NIH 80-23), the Association for Research in Vision and Ophthalmology Statement for use of Animals in Ophthalmic and Vision Research and were approved by the Augusta University Institutional Animal Care and Use Committee. Male C57BL/6J mice of different age groups obtained from a commercial vendor (Jackson Laboratories, Bar Harbor, ME, USA) and the National Institute of Aging (National Institutes of Health, Bethesda, MD, USA) were housed under identical conditions in a pathogen-free environment with a 12:12 h light/dark cycle and free access to laboratory chow and water. Mice were acclimatized for at least 1 week before the experiments.

### Cell culture

Human retinal pigment epithelial (ARPE-19) cells were cultured in Dulbecco’s modified Eagle medium DMEM/F12 medium (supplemented with 10% fetal bovine serum, 100 U/ml penicillin, and 100 μg/ml streptomycin) and maintained at 37 °C in a humidified chamber with 5% CO_2_. The culture medium was replaced with fresh medium every other day. Cultures were passaged by dissociation in 0.05% (w/v) trypsin in phosphate-buffered saline (PBS; 0.01 M phosphate buffer, 0.0027 M KCl, 0.137 M NaCl, pH 7.4). Completely confluent, well-differentiated cultures were used for experimentation.

Primary mouse RPE cells were prepared from C57BL/6J mouse eyes and the purity of these cultures was confirmed per our published method [50–53]. Primary mouse RPE cells were then maintained at 37 °C in a humidified chamber with 5% CO_2_ and sub-cultured using trypsin–EDTA solution. All experiments were carried out using primary RPE cells in passages 1 and 2.

### Reverse transcription–quantitative polymerase chain reaction

Total RNA was isolated from RPE/eyecup of mice (2, 12 and 18 months old) or ARPE-19 cells using RNAeasy mini kit (Qiagen, USA). cDNA was prepared from total RNA using the iScript cDNA Synthesis Kit (Bio-Rad) and subjected to qPCR assay. Assays were performed in 96-well PCR plates using All-in-One™ qPCR Mix (Genecopia, USA). The reaction volume of 20 μl contained 10.0 μl SYBR green master mix (2X), 1 μl cDNA, 1 μl of each primer and 7 μl nuclease-free water. Primer sequences are listed in Table 1. The following two-step thermal cycling profile was used (StepOnePlus Real-Time PCR, Life Technologies, Grand Island, NY): Step I (cycling): 95 °C for 5 min, 95 °C for 15 s, 60 °C for 30 s and 72 °C for 15s for 40 cycles. Step II (melting curve): 60 °C for 15 s, 60 °C 1 min and 95 °C for 30 s. The template amplification was confirmed by melting curve analysis. mRNA expression of genes were normalized to 18s expression and fold change in expression was calculated by 2^–∆∆Ct^ method.

**Table 1:** Primer sequences used for qPCR.

**Gene**	**Forward (5’-3’)**	**Reverse (5’-3’)**
*Homo sapiens*		
P21Waf/cip1	AAGTCAGTTCCTTGTGGAGC	GCCATTAGCGCATCACAGTC
CTGF	ACATTAAGAAGGGCAAAAAGTGC	GTGCAGCCAGAAAGCTCAAA
APOJ	AGGGACTGTCATACCAGTGA	TTGTCGCACCTTGGTCAGAA
IL-6	CTCAATATTAGAGTCTCAACCCCCA	TGTTACATGTTTGTGGAGAAGGAG
IL-8	GCTCTGTGTGAAGGTGCAGTT	ACCCAGTTTTCCTTGGGGTC
18S	CCCGTTGAACCCCATTCGT	GCCTCACTAAACCATCCAATCGGTA
*Mus musculus*		
*Nmnat*	CCTTCAAGGCCTGACAACAT	ACCGACCGGTGAGATAATGC
*Nampt*	AACCAATGGCCTTGGGGTTA	TAACAAAGTTCCCCGCTGGT
*Qprt*	GTGGAATGTAGCAGCCTGGA	TGCAGCTCCTCAGGCTTAAA
*18s*	CCAGAGCGAAAGCATTTGCCAAGA	AGCATGCCAGAGTCTCGTTCGTTA

### FK866 and Nicotinamide mononucleotide (NMN) treatments

ARPE-19 cells were serum starved overnight and treated with varying doses (0.01-10μM) of FK866 (Sigma-Aldrich) for different time intervals (24, 48 or 72 h). Following FK866 treatment, cells were collected for one or more of the following analyses: MTT assay, NAD^+^ measurement, SIRT1 activity assay, RNA or protein studies. Primary mouse RPE cells were treated and analyzed similarly except that lower doses of FK866 (0.01, 0.1 and 0.5 μM) were employed. The above experiments were used to establish the optimal dose and time for use of FK866 to adequately inhibit NAMPT without inducing robust cellular toxicity in ARPE-19 and primary RPE cells. Congruent with these studies, ARPE-19 and primary mouse RPE cell cultures were exposed to FK866 (10 μM) for 72 h in the presence or absence of varying concentrations of the NAD^+^ precursor, nicotinamide mononucleotide (NMN; 0.05 – 1.0 mM).

For *in vivo* studies, adult male C57BL/6J mice were deeply anesthetized via a single intraperitoneal injection of ketamine (80 mg/kg) and xylazine (12 mg/kg; Sigma-Aldrich, St. Louis, MO, USA). Upon confirmation of anesthetic depth via toe pinch, FK866 (10μM) was delivered sub-retinally in a total volume of 2 μL using a 33-gauge Hamilton Syringe (Hamilton, Reno, NV, USA) at day 0 and again at day 7. Sham control eyes (left eyes) were injected in the same manner except with 2 μL of 0.01M PBS. A group of FK866 injected mice were treated with 150 mg/kg NMN (*i.p.*) once daily for 2 weeks [36]. Mice were euthanized one-week post delivery of the second FK866 injection and RPE tissue harvested for measurement of NAD^+^ and protein analyses.

### Western blot analysis

Protein was extracted from RPE/eyecup of mice (2, 12 and 18 months old) or ARPE-19 cells or primary RPE cells using RIPA cell lysis buffer (Thermo Scientific) containing protease and phosphatase inhibitors and concentration was determined using the coomassie protein assay reagent (Sigma-Aldrich, USA). Equivalent amount of protein samples were subjected to SDS–PAGE, transferred to PVDF membranes, and then incubated with primary antibodies: SIRT-1, p21^Waf/Cip1^ (1:1000; Cell signaling) and p16^INK4a^ (1:500; Abcam) overnight at 4°C. Next day, blots were washed with TBST and incubated with horseradish peroxidase conjugated secondary antibody (1:3000; Sigma-Aldrich, USA) for 60 min with gentle shaking at room temperature. Blots were then washed (with TBST) and developed with chemiluminescence reagent (Bio-Rad, Hercules, CA) using autoradiography films (Genesee Scientific, San Diego, CA). β-actin (1:3000; Sigma-Aldrich, USA) expression was evaluated to determine equivalent loading. Scanned images of blots were used to quantify protein expression using NIH ImageJ software (http://rsb.info.nih.gov/ij/).

### NAD^+^ assay

NAD^+^ levels in cells and tissue were quantified with a commercially available kit (Sigma, MO, United States) according to the manufacturer's instructions.

### SIRT1 activity assay

The SIRT-1 deacetylase assay in ARPE-19 cells was performed using a fluorometric SIRT1 assay kit (Sigma-Aldrich, USA), according to the manufacturer’s protocol. Briefly, the reaction was carried out at 37°C for 30 min and deacetylase activity was detected as a fluorescence emission at 450 nm, using an excitation wavelength of 360 nm. The intensity of the fluorescence emitted by the compounds at 450 nm was subtracted from initially determined assay values.

### β-galactosidase assay

Senescence-associated beta-galactosidase (β-Gal) staining was performed according to the manufacturer's protocols (Cell Signaling Technology). At the end of treatments, cells were fixed for 15 minutes in 1x fixative solution (formaldehyde-glutaraldehyde mix), followed by PBS washes, and incubated overnight with β-Gal staining solution in a dry incubator at 37°C. Cells were viewed under brightfield microscopy (Leica BM5500B Microscope; Leica Biosystems, Wetzlar, Germany) for blue color development and were photographed using a Photometrics CoolSNAP HQ^2^ camera and associated Leica Application Suite software v4.1.

### CellROX and MitoSOX staining

ARPE-19 cells were treated with 10 μM FK866 for 72 hr. as mentioned above. At the end of treatments, cells were incubated for 5 μM CellROX for 30 min or 5 μM MitoSOX for 10 min. At the end of incubation, cells were washed with PBS, mounted using fluoroshield mounting medium with DAPI and images were captured using Zeiss Axioplan-2 imaging florescence microscope.

### Statistical analysis

Results are presented as mean ± S.E.M for minimum of three independent experiments. Statistical significance was defined as p < 0.05 and determined using student’s t-test (normally-distributed data). Graphs were prepared using GraphPad Prism 7 software.

## Supplementary Material

Supplementary File
