# Supplementary material for: Loss of NAMPT in aging retinal pigment epithelium reduces NAD+ availability and promotes cellular senescence
Source: Aging (Albany NY). 2018 Jun 12;10(6):1306–23. doi: 10.18632/aging.101469 (PMC6046249; doi:10.18632/aging.101469)
Supplement: Supplementary File [file aging-10-101469-s001.pdf]

SUPPLEMENTARY MATERIAL

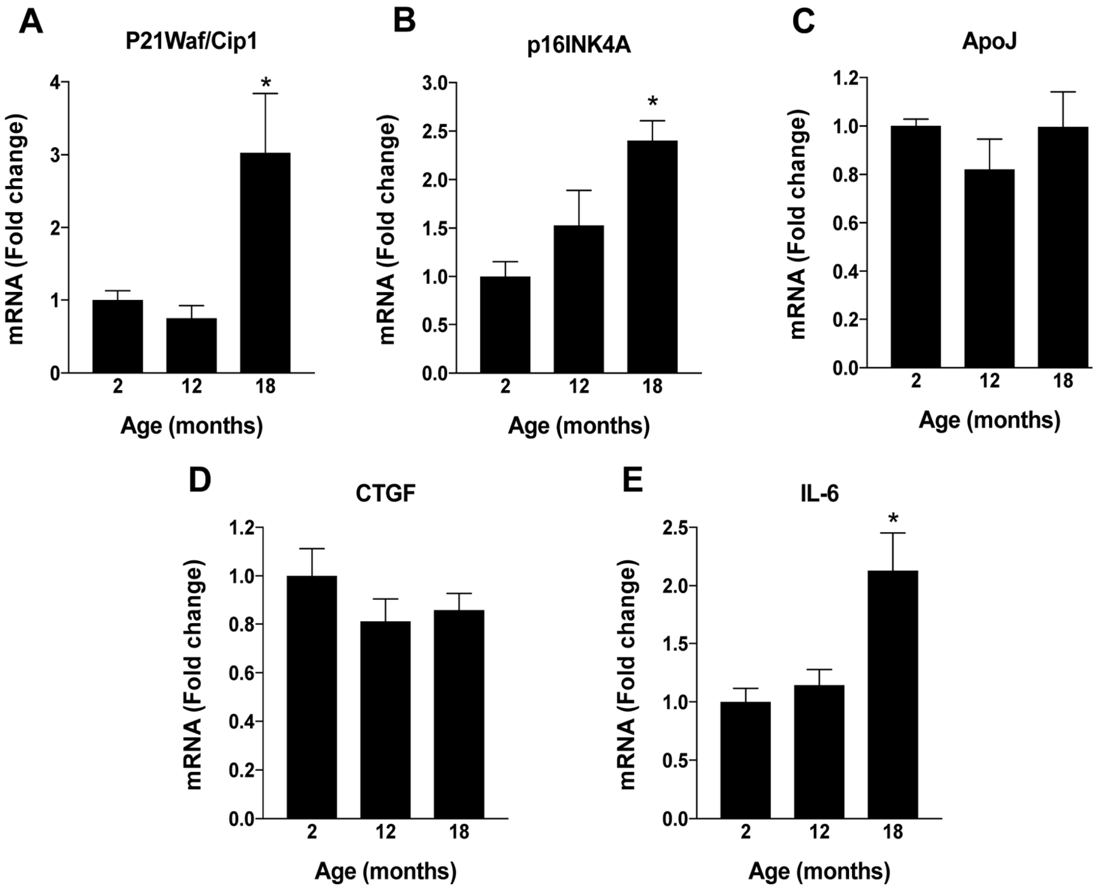

Figure S1. Changes in RPE senescence markers with aging in mice. RPE/eye cup was dissected from 2, 12 and 18 months old male C57BL/6J mice to evaluate changes in the expression of various senescence markers. (**A**-**E**) Changes in mRNA expression of markers associated with senescence were performed by qPCR. Data is presented as mean ± S.E.M for n=5. mRNA expression of genes were normalized to 18s expression. *p<0.05 compared to 2M (two months old mice).

Table S1. Primer sequences used for qPCR.

Mus musculus		
p21Waf/cip1	TATCCAGACATTCAGAGCCAC	CGAAGTCAAAGTTCCACCGT
p16ink4a	TCTTGGTCACTGTGAGGATTCA	GAACGTTGCCCATCATCATCA
Ctgf	TCCACCCGAGTTACCAATGA	TGCACTTTTTGCCCTTCTTA
ApoJ	TAGGCTTCCAGAAAGCTCCT	ATAGCGCTCTGCTCAAGTACA
IL-6	CTTCTGGAGTACCATAGC	TCTGTTAGGAGAGCATTG
18s	CCAGAGCGAAAGCATTTGCCAAGA	AGCATGCCAGAGTCTCGTTCGTTA
